# Collaborative learning from distributed data with differentially private synthetic data

**DOI:** 10.1186/s12911-024-02563-7

**Published:** 2024-06-14

**Authors:** Lukas Prediger, Joonas Jälkö, Antti Honkela, Samuel Kaski

**Affiliations:** 1 Aalto University, Espoo, 00076 Finland; 2 University of Helsinki, Helsinki, 00014 Finland; 3 University of Manchester, Manchester, M13 9Pl UK

**Keywords:** Collaborative learning, Differential privacy, Health informatics, Synthetic data

## Abstract

**Background:**

Consider a setting where multiple parties holding sensitive data aim to collaboratively learn population level statistics, but pooling the sensitive data sets is not possible due to privacy concerns and parties are unable to engage in centrally coordinated joint computation. We study the feasibility of combining privacy preserving synthetic data sets in place of the original data for collaborative learning on real-world health data from the UK Biobank.

**Methods:**

We perform an empirical evaluation based on an existing prospective cohort study from the literature. Multiple parties were simulated by splitting the UK Biobank cohort along assessment centers, for which we generate synthetic data using differentially private generative modelling techniques. We then apply the original study’s Poisson regression analysis on the combined synthetic data sets and evaluate the effects of 1) the size of local data set, 2) the number of participating parties, and 3) local shifts in distributions, on the obtained likelihood scores.

**Results:**

We discover that parties engaging in the collaborative learning via shared synthetic data obtain more accurate estimates of the regression parameters compared to using only their local data. This finding extends to the difficult case of small heterogeneous data sets. Furthermore, the more parties participate, the larger and more consistent the improvements become up to a certain limit. Finally, we find that data sharing can especially help parties whose data contain underrepresented groups to perform better-adjusted analysis for said groups.

**Conclusions:**

Based on our results we conclude that sharing of synthetic data is a viable method for enabling learning from sensitive data without violating privacy constraints even if individual data sets are small or do not represent the overall population well. Lack of access to distributed sensitive data is often a bottleneck in biomedical research, which our study shows can be alleviated with privacy-preserving collaborative learning methods.

**Supplementary Information:**

The online version contains supplementary material available at 10.1186/s12911-024-02563-7.

## Introduction

Often access to the data needed for the most crucial statistical inference tasks is strictly limited to protect the privacy of data subjects. One example where this is especially prevalent is the case of medical data. Due to this limitation, such data cannot be easily combined across different origins to make population level statistical discoveries. This can be a severe problem during newly developing situations such as epidemics, in which data at each single origin is initially scarce and such discoveries are essential in making informed population level decisions, for example regarding the measures to take to prevent infectious diseases from spreading. Recent advances in generative models as well as privacy-preserving machine learning make sharing synthetic data an appealing solution to mitigate the privacy concerns of sharing sensitive data.

**Fig. 1:**
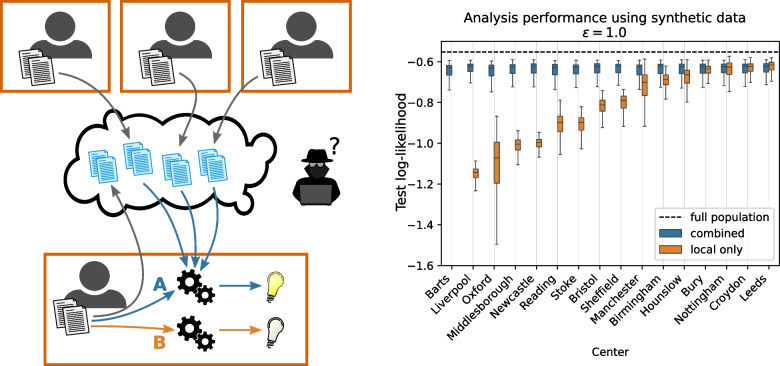
**Left:** Schematic overview of our setup. Multiple parties create synthetic data replicas of their local data under privacy guarantees and make them publicly available. Any single party can then use the published synthetic data when performing a data analysis task (case A, blue) to improve results over only using their local data (case B, orange). The original data never crosses the (orange) privacy barriers. **Right:** Predictive log-likelihoods of the learned model (blue) are significantly improved over using only locally available data (orange) for most parties (centers). Uncertainty is also reduced. The dashed black line shows the log-likelihood for an impractical ideal setting where the analysis could be performed over the combined data of all parties. Log-likelihood is evaluated on a held-out test set of the whole population and normalised by dividing with the size of the test set. The box plots show the distributions of log-likelihood for parameters sampled from the distributions implied by maximum-likelihood solution and errors obtained from the analysis task and over 10 repeats of the experiment. Boxes extend from $25\%$ to $75\%$ quantiles of the obtained log-likelihood samples, with the median marked in the box. Whiskers extend to the furthest sample point within 1.5 inter-quartile range. Higher mean log-likelihoods of combined over local only are statistically highly significant ($p < 0.001, n_{\text {local only}}= {1\,000}, n_{\text {combined}} = {100\,000}$) for all centers except *Nottingham*, *Croydon* and *Leeds*. Local data log-likelihood of outlier center *Barts* is cut off for improved readability (median: $-3.65$). The full figure can be found in Fig. S3

Releasing synthetic data generated from a model trained with differential privacy [1] has been proposed previously as a way to enable privacy-preserving sharing of sensitive data sets [2–12]. These previous works have focused on methods for releasing a synthetic data set for a single sensitive data set. On the other hand, other recent work raised concerns that using such synthetic data from a single source as if it was original data may have detrimental effects [13]. However, the usefulness of combining several of such synthetic data sets released by multiple parties for collaborative analysis has not been studied before. This is particularly true for the case where data are not homogeneous amongst the parties and each party holds a relatively small sensitive data set. We bridge this gap in the present work by performing a case study on a real world data set.

Throughout this work we consider a setting in which there exist $M \ge 2$ parties that are interested in performing statistical analyses over a population. Each party *m* has access to a local data set $D_m$ that are *disjoint* and *non-uniformly* sampled from the overall population: Every $D_m$ may follow a distribution shifted away from that of the overall population (i.e., $\Pr [x | m] \ne \Pr [x]$). We assume that the parties cannot simply pool their data to perform the analysis due to the sensitive nature of the data, consisting, e.g., of electronic health records. Instead, we suggest that each party trains a generative model on their local data using privacy-preserving machine learning techniques and publishes synthetic data sampled from this model in place of the sensitive data. The party then obtains similar synthetic data from other parties, which it combines with its own local data before performing its analysis task. This process is depicted in Fig. 1. The distributed data setting we consider is similar to that of (cross-silo) federated learning (FL) [14, 15] but does not require the parties to engage in a centrally coordinated joint computation and is not limited to computing parameters for a single model. Instead, parties generate synthetic data completely locally, publishing the results. Each party then downloads the published synthetic data sets and locally combines those with its own data before to perform its analysis task over population (instead of only local) data. See Section Federated learning for further discussion on differences and relative merits to FL.

For training the generative model we adopt the formal framework of differential privacy (DP), which guarantees that the obtained generative model would be essentially identical if any data subject were to be removed from a party’s local data set. Since the parties’ local data sets are disjoint, these privacy guarantees hold independently for all synthetic data sets. Consequently the framework we describe achieves DP in the billboard model [16], i.e., the outcome of the analysis task performed by each party is differentially private with respect to the data from all other parties. The formal privacy definition and generative model are detailed in Section Methods.

Summarising, in our setting we assume that

1. the parties’ local data sets are disjoint,
2. the parties’ combined data accurately represent the overall population but any particular party’s local data may be arbitrarily skewed,
3. each party’s goal is to optimise their analysis on the population level (not only their local data),
4. the results of a party’s analysis are kept private,
5. a party that receives synthetic data will also share synthetic data of their own data, and,
6. parties are non-malicious, i.e., they do not actively try to negatively affect other parties’ performance.

This setting leads to an apparent dilemma: If the local data of a party *m* is not sufficient to learn the analysis model for the global population well, this suggests that it might not be possible to learn a good generative model from it either, especially under privacy constraints. Hence, we should expect that in the case where most parties only have access to small data sets and would therefore be interested in obtaining additional data to improve their analysis, the synthetic data sets shared amongst the parties might not be of sufficient quality to actually help. Specifically, the question arises: Does incorporating (low-fidelity) synthetic data generated from small data sets of other parties improve results of the analysis performed by party *m* over just using its own (small) local data set? We answer this question in the affirmative.

Concretely, in our setting and under the assumptions stated above, we empirically demonstrate on a real-world health data set from the UK Biobank [17] that:

1. Complementing local data with synthetic data of similarly sized data sets consistently increases the utility in the analysis task, and this increase can be drastic.
2. This effect is more pronounced on smaller local data.
3. As the number of parties sharing data increases, the results from the analysis on combined data quickly approach those obtained from the overall population.
4. Parties suffering from local skew benefit from sharing, even if their local data is comparatively larger than that of any other party. Such skew can, for example, arise from underrepresented minority groups in a party’s local data.

The remainder of the paper is organised as follows: We first present the data set and methods applied in our case study in Section Methods. We then present the results of our empirical study in Section Results, followed by a discussion of the results and related literature and concluding remarks in Section Discussion and Conclusions respectively.

## Methods

### Differential privacy

Our setting relies on generating synthetic data with quantifiable privacy guarantees provided by the framework of *differential privacy* (DP) [1]. DP is considered a standard definition for privacy and is formally defined as the following property of an algorithm:

#### Definition 1

**(**$(\varepsilon , \delta )$ **Differential Privacy** [1]**)** For $\varepsilon \ge 0$ and $\delta \in [0,1]$, a randomised mechanism $\mathcal {M}$ satisfies $(\varepsilon ,\delta )$ differential privacy if for any two data sets different in only one element, $D, D^{\prime } \in \mathcal {D}$, and for all sets of outputs $S \subseteq \text {im}(\mathcal {M})$, the following constraint holds:

1 $$\begin{aligned} \Pr (\mathcal {M}(D) \in S) \le e^{\varepsilon } \Pr (\mathcal {M}(D^{\prime }) \in S) + \delta . \end{aligned}$$

The above definition is also known as *approximate* DP. A stricter definition requiring $\delta =0$ is known as *pure* DP. In this work we focus on the former.

We consider the so-called *add-remove* neighbourhood relation (otherwise known as *unbounded DP* [18]), i.e., $D^{\prime }$ can be obtained from *D* by adding or removing a single data sample at any party, which act as trusted aggregators towards the individual data subjects.

Intuitively, the effect that removal or addition of any individual data item in the inputs of a DP algorithm has on the output is limited by the privacy parameters $\varepsilon$ and $\delta$. Lower values for these parameters correspond to stricter privacy as they force the output distributions for different inputs to be more similar. In the extreme case of $\varepsilon = 0$ and $\delta = 0$, the output of $\mathcal {M}$ would need to be independent of the inputs. We stress that DP is not a single specific technique applied to anonymise data but rather the formal property of an algorithm that provides measurable privacy guarantees. Privacy guarantees provided by DP hold irrespective of auxiliary information available to an attacker. In contrast, it has been shown that straightforward anonymisation techniques on the data set, such as removing or masking certain attributes, in general do not satisfy DP and are vulnerable to re-identification attempts [19].

An important property of differential privacy is *composability*, which allows to split privacy parameter $\varepsilon$ across multiple invocations of a DP mechanism while guaranteeing that the resulting combined algorithm (that combines all so obtained outputs) still satisfies ($\varepsilon , \delta$)-DP. In that sense $\varepsilon$ is often thought of as a *privacy budget* that can be split over subsequent steps in a privacy-preserving analysis. Another important property is *post-processing immunity*, which guarantees that any processing of the outputs of a DP algorithm $\mathcal {M}$ with a function *f* is still private in the DP sense, i.e., the composition $f \circ \mathcal {M}$ is also $(\varepsilon , \delta )$-DP.

Under our assumptions stated in the Introduction, it follows that our framework seen as a whole, which results in each party obtaining a local statistical model of the data of all parties, satisfies *billboard differential privacy* [16, 20]. This ensures that any party’s model is $(\varepsilon , \delta )$-DP with respect to all other parties’ data.

#### Definition 2

**(**$(\varepsilon , \delta )$ **Billboard DP** [20]**)** Let $\mathcal {M}(D) = [f_m(D_m, g(D))]_{m=1,\ldots ,M}$ be the output of a randomised mechanism $\mathcal {M}$ with input $D =\bigcup _{m=1}^M D_m \in \mathcal {D}$ where $f_m: \mathcal {D}_m \times \mathcal {Q} \rightarrow \mathcal {R}_m$ and $g: \mathcal {D} \rightarrow \mathcal {Q}$. For $\varepsilon \ge 0$ and $\delta \in [0,1]$, $\mathcal {M}$ satisfies $(\varepsilon , \delta )$ billboard differential privacy if for any *m* and any two sets $D_m$ and $D^{\prime }_m$, different in only one element, and for all sets of outputs $S \subseteq \mathcal {R}_{-m} = \bigcup _{j\in \{1,\ldots ,M\}\setminus \{m\}} \mathcal {R}_j$, the following constraint holds:

2 $$\begin{aligned} \Pr (\mathcal {M}(D)_{-m} \in S) \le e^{\varepsilon } \Pr (\mathcal {M}(D^{\prime })_{-m} \in S) + \delta , \end{aligned}$$

where $\mathcal {M}(\cdot )_{-m}$ denotes the output vector of $\mathcal {M}$ with the *m*-th element removed.

### Differentially private data sharing

Several methods for the technical implementation of DP data sharing via synthetic data were previously proposed [2–12]. On a high-level, they all specify a $(\varepsilon , \delta )$-DP algorithm $\mathcal {T}_{G}$ for training a generative model *G* from the sensitive input data and then sample synthetic data from *G*. Due to the post-processing property of DP, the information leakage through the synthetic data is then guaranteed to be bounded by the privacy parameters $\varepsilon$ and $\delta$, regardless of the number of samples drawn from *G*.

In this work we are not suggesting any new inference algorithms for DP data sharing. Instead we investigate a general approach for collaborative learning based on synthetic data, for which any of the aforementioned data generating methods can be used. This allows expert users to choose the most appropriate model and algorithm for describing their data in order to improve the quality of the shared synthetic data. The particular method and model we used for our experiments are described in the following sections.

### UK Biobank SARS-CoV-2 data set

To replicate a plausible application scenario for the analysis task, we follow a study performed by Niedzwiedz et al. [21] and use a data set obtained from the UK Biobank [17] in our experiments. The data consists of five ethnic and socioeconomic factors, among them e.g. an individual’s ethnicity and education level, all of which can be considered sensitive information.^1^ All the features are categorical. They were surveyed during voluntary sign up for inclusion in the UK Biobank cohort in one of 22 *assessment centers*. We restricted the data set to individuals for which at least one SARS-CoV-2 test result was present (before 2021-03-15) for a remaining total of ${58\,253}$ records split over 16 assessment centers. These splits range in size from ${1\,867}$ to ${5\,922}$ records, with a median of ${3\,729}$.^2^ We use the assessment centers as parties with their respective split of the full UK Biobank cohort as local data sets $D_m$. For most of our experiments we additionally subsample each center’s data to 10% of its initial size to create data that is sufficiently small so that no single center can learn the regression task well.

### Models for analysis task and synthetic data generation

Following [21], we formulate as our analysis task *f* a Poisson regression model which predicts the likelihood of a positive test for a SARS-CoV-2 infection based on the ethnic and socioeconomic factors (referred to as *analysis model* in the following). Formally we label the vector of regressors, i.e., the features used for prediction, as $\varvec{x}$, the SARS-CoV-2 test result as *y* and the regression parameters as $\varvec{w}$ and obtain *f* as:

3 $$\begin{aligned} {f}(\varvec{x}; \varvec{w}){} & {} = \arg \max _{y \in \{0,1\}} \frac{\lambda ^y e^{-\lambda }}{y!},\end{aligned}$$

4 $$\begin{aligned} \lambda{} & {} = e^{\varvec{w}^T \varvec{x}}. \end{aligned}$$

We use the statsmodels [22] Python package to obtain parameter and corresponding standard error estimates.

For synthetic data generation we consider a parametric probabilistic model consisting of two parts. One part exactly mirrors the Poisson regression of the analysis task and models the SARS-CoV-2 test results based on the regressors. The other part is a mixture model which models the regressors following Jälkö et al. [11]. We use 16 mixture components based on empirical tuning of the model’s hyperparameters. Formally, the generative model ${G}_m$ for each party is:

5 $$\begin{aligned}{} & {} p(\varvec{x} \mid \varvec{\theta }_{\varvec{x}}, \varvec{\pi }) = \sum \limits _{r=1}^{16} \varvec{\pi }_r \prod \limits _{j=1}^d p(\varvec{x}_j \mid \varvec{\theta }_j^{(r)})\end{aligned}$$

6 $$\begin{aligned}{} & {} p(y \mid X, \varvec{w}) = \frac{\lambda ^y \exp (-\lambda )}{y!}. \end{aligned}$$

Here $p(\varvec{x}_j \mid \varvec{\theta }_j^{(r)})$ is the categorical distribution over values of the *j*-th feature of $\varvec{x}$ in mixture component *r* with parameters $\varvec{\theta }_j^{(r)}$ and $\varvec{\pi }$ is a vector of mixture coefficients. In our experiments we consider $d=5$ categorical features.

To infer an approximate posterior over the parameters of the statistical models for each party, we use the differentially private variational inference algorithm (DPVI) [23, 24] (based on the implementation in d3p [25]) as $\mathcal {T}_{G}$ with $\varepsilon = 1$ and $\delta = 10^{-6}$ in all experiments. Synthetic data sets (of the same size $N_m$ as the party’s local data set) are sampled from the inferred model for each party.

DPVI is variational inference algorithm using DP stochastic gradient descent [26–28]. It achieves privacy by clipping the gradients to a maximum norm (to limit the maximum effect of the update) and adding Gaussian noise calibrated to the desired privacy parameters in each update step. It relies on the composition property of DP to guarantee privacy over all iterations and we compute the total privacy budget over all iterations using the Fourier Accountant [29, 30].

### Sharing multiple synthetic data sets

To quantify additional uncertainty introduced by sampling a finite data set from the generative models we follow an approach suggested by Räisä [12]: Each party performs *K* repetitions of sampling, publishing and training with synthetic data. In each sampling repetition, the party draws a parameter vector from the learned posterior and then samples data according to the generative model outlined above. Each of the *M* parties thereby receives $(M-1)K$ synthetic data sets and combines them into *K* combined sets on which it performs the analysis task. After this, each party locally combines the resulting *K* analysis models, by either distilling a single combined model out of them or setting up a suitable ensemble [12, 31]. Following [12], we use Rubin’s rules to combine the obtained parameter and standard error estimates into a single model, analogous to the concept of multiple imputation [32, 33], where missing data is repeatedly replaced with resampled available data. For each of the $k \in [K]$ synthetic data sets, we fit the downstream Poisson regression model and obtain the regression coefficients $\textbf{w}_k$ and corresponding variances $\textbf{v}_k$. Rubin’s rules [34] estimate the posterior of the regression weights as a normal distribution with mean^3^$\overline{\textbf{w}}_K = \frac{1}{K}\sum \nolimits _{k=1}^K \textbf{w}_k$ and variance $\hat{\textbf{v}}_K^{(-)} = (1 + K^{-1}) \textbf{b} - \overline{\textbf{v}}$ where

7 $$\begin{aligned} \textbf{b}{} & {} = \frac{1}{K-1} \sum \limits _{k=1}^K (\textbf{w}_k-\overline{\textbf{w}}_K)^2,\end{aligned}$$

8 $$\begin{aligned} \overline{\textbf{v}}{} & {} = \frac{1}{K} \sum \limits _{k=1}^K \textbf{v}_k. \end{aligned}$$

Note that $\hat{\textbf{v}}_K^{(-)}$ can be negative. When this occurs, we use a more conservative alternative of Rubin’s rules [33] and set variance to $\hat{\textbf{v}}^{(+)}_K = (1 + K^{-1}) \textbf{b} + \overline{\textbf{v}}$. We denote the resulting final variance estimate with $\hat{\textbf{v}}_K$ We set the number of synthetic data sets sampled by each party as $K=100$.

### Evaluation metrics

In order to assess the quality of the synthetic data, we evaluate the analysis model *f* learned by each party on a *global test set* $D_{\text {all,test}}$ representing the full cohort. $D_{\text {all,test}}$ is obtained by splitting each center’s local data into training and test sets with a 80/20 (training/test) ratio, then taking the union over the local test sets to obtain the global test set: $D_{\text {all,test}} = \bigcup _m D_{m,\text {test}}$.^4^ We use the predictive log-likelihood of *f* with learned parameters $\varvec{w}$ as our measure for utility:

9 $$\begin{aligned} u({f}(\cdot ; \varvec{w})) = \sum \limits _{(\varvec{x}, y) \in D_{\text {all,test}}} y\ln \lambda - \lambda - \ln (y!), \end{aligned}$$

with $\lambda$ as defined in Eq. (4).

We use a Monte Carlo approach to sample a distribution of log-likelihoods: First we approximate the distribution of parameter estimates by a diagonal Gaussian fully described by the mean $\overline{\textbf{w}}_K$ and variance $\hat{\textbf{v}}_K$ from Rubin’s rules. We then sample parameter vector $\varvec{w}$ from this distribution and compute the corresponding log-likelihood for $\varvec{w}$ on $D_{\text {all,test}}$. We repeat this sampling 100 times. The sampled log-likelihood distribution then summarises the overall performance of the learned analysis model across all parameters as well as the estimated standard errors.

We further repeat all experiments 10 times with different seeds for internal randomness, beginning with the inference of the generative models for shared synthetic data. We additionally sample 100 random permutations of the order in which data from other parties becomes available. The plots shown throughout the Results section always show the distributions of log-likelihood results over all repetitions, for a total of ${100\,000}$ samples.

Whenever significance on differences between means of the obtained log-likelihood sample sets were reported, Welch’s t-test [35] was used after ranking the tested samples to account for unequal variances between, as well as non-uniformity within, each sample set, following [36]. One-sided tests were used in all cases except for Figs. 4 and 5, which used two-sided tests.

## Results

### Data sharing consistently improves results over using local data only

We first show that a party *m* which incorporates synthetic data shared by all other parties improves the performance of its analysis.

The right side of Fig. 1 shows the log-likelihood of the Poisson regression analysis model on the global test set for each center fitting the model only on its locally available data (orange) compared to incorporating synthetic data shared by all other centers (blue). Across most centers (subsampled to $10\%$ of the original size^5^) we observe a clear improvement in average predictive log-likelihood when pooling synthetic data, as well as a reduction in spread, with the exception of *Nottingham*, *Croydon* and *Leeds*. That is, we consistently obtain models that perform better and exhibit significantly less standard error in parameters. The log-likelihood distributions we obtain from including synthetic data are close to an ideal, privacy-agnostic baseline where we could simply pool all centers’ data before performing our regression (black dashed line); this situation is precluded in practice due to privacy constraints.

The results appear consistent across centers even though the statistical signal for the analysis model present in the local data varies drastically across the centers. This is true despite some of the other participating centers contributing data which alone result in a very poor model for predictions on the global population (such as e.g. *Barts* and *Oxford*). Centers whose local data already allow fitting an analysis model that performs quite well on the population level test set, such as e.g., *Nottingham*, *Croydon* and *Leeds*, may not see all the same benefits if the privacy parameters are too strict, as is the case here. However, as we will see later, they may still benefit from incorporating only a portion of the synthetic data shared by other centers, so that participating might still be worthwhile for them.

### Gains increase quickly with number of shared data sets

We have seen in the previous section that almost every center improves the performance of its analysis task by incorporating large amounts of synthetic data from different sources. Indeed, due to the large amount of synthetic data available, the effect of the local data on the outcome of the analysis appears to be quite limited in that setting. It is now natural to ask how soon these improvements manifest, i.e., how much synthetic data is required. We investigate this in the following experiment: We fix a center and add synthetic data from other centers one by one, then evaluate the log-likelihood at every step. We repeat this experiment 100 times for different sequences in which synthetic data from other centers is added.

**Fig. 2:**
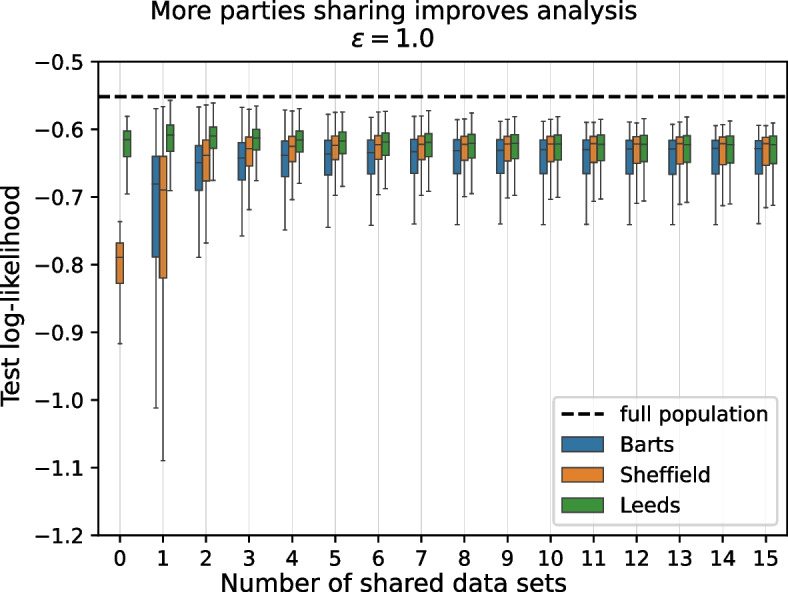
The log-likelihood of the learned analysis model improves rapidly as synthetic data from other centers becomes available. Spread in log-likelihoods may initially increase when only a few synthetic data sets are incorporated, but then diminishes rapidly with the number of additional data sets. The dashed black line shows the log-likelihood for an ideal setting where the analysis could be performed over the combined data of all parties. 10 repeats of the experiment, each with 100 repeats with different orders in which synthetic data is added. The improvements in mean log-likelihood between subsequent steps for all centers except *Bristol*, *Bury*, *Croydon*, *Hounslow*, *Leeds* and *Nottingham* are highly significant ($p < 0.001, n={100\,000}$) up to five centers releasing synthetic data. Local results for outlier center *Barts* (median: $-3.65$) cut off for readability. See Table S4 for corresponding *p*-values, and Fig. S4 for the full figure

Figure 2 shows box-plots of the log-likelihood distributions as more and more other centers make synthetic data available, from the perspective of centers *Barts*, *Sheffield* and *Leeds*, representing respectively centers with bad, intermediate and good fit when using only local data (see Fig. 1, right). Results for the remaining centers are consistent with the ones shown here and can be seen in Fig. S4.

We see that for *Barts* and *Sheffield* the median log-likelihood improves quickly from the first step of adding synthetic data from a single source, but its spread may increase initially, as is the case for *Sheffield*. Spread then diminishes quickly as more synthetic data becomes available. After about five steps no further improvements occur. *Leeds* initially shows an improvement when incorporating only small amounts of additional synthetic data (from one or two other sources) but experiences a drop on utility as the lower quality synthetic data overwhelms it’s good local data signal.

### Data sharing helps especially when local data sets are small

We now turn to investigating the effect of the size of the locally available data. Learning of many machine learning models becomes less reliable with smaller amounts of data and the data sharing approach requires each party to learn a generative model under privacy constraints, which poses additional limitations to learning. It is natural to ask whether the quality of the synthetic data released by the parties deteriorates more quickly than that of the analysis model trained only on local data as the number of data points decreases.

**Fig. 3:**
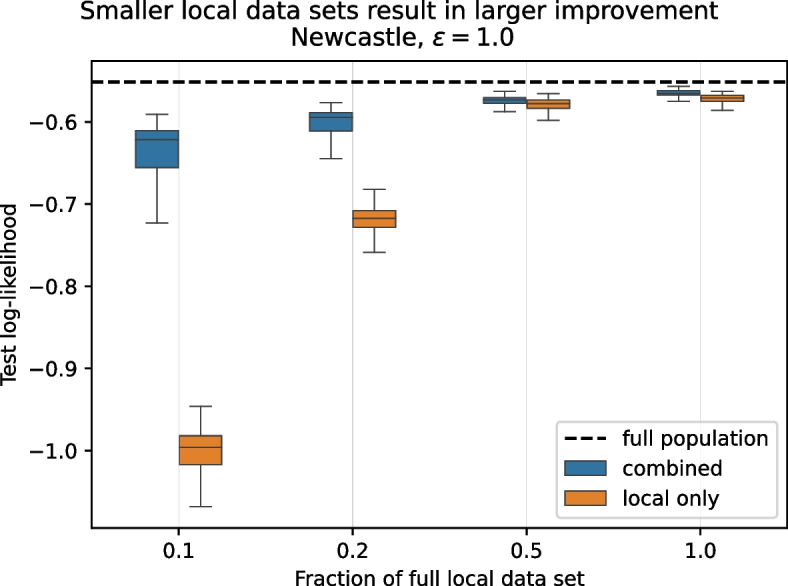
Usefulness of sharing of data using synthetic data sets is retained in the small data regime: Performance of a model trained including shared synthetic data from other parties (blue), *all with similarly small local data*, decreases much less than that of a model trained only on locally available data (orange). Higher mean log-likelihoods of combined over local only are statistically highly significant ($p < 0.001, n_{\text {local only}}={1\,000}, n_{\text {combined}}={100\,000}$) for all data set sizes

To investigate this, in addition to the $10\%$ subsampling used in the earlier experiments we subsample the training data to $20\%$, $50\%$, $100\%$ (i.e., no subsampling) of the original number of samples before running the data sharing procedure, which we again repeat ten times for each setting. Figure 3 shows the predictive log-likelihood distribution of training the analysis model only on local data (orange) and after including the shared data (blue) for the different amounts of data (at all centers) for the *Newcastle* assessment center, for which the largest amount of local data is available.

We observe that, as the local data gets smaller, the performance of the model trained on local data only deteriorates much faster than that obtained using the data sharing approach. This strongly suggests that the positive effect of getting additional data for the analysis task outpaces the negative effects smaller local data has on learning the generative models. This is most likely due to the negative effects of the latter being mitigated by a sufficient amount of parties sharing data: Even when the individual sets are small and of poor quality, in combination they still carry an overall strong enough signal to enable meaningful analysis. The results for other centers are consistent with this.

### Parties can correct for skew in their local distribution

The final remaining question of interest is whether a large party (i.e., a party with a large amount of local training data) gains anything from engaging in the data sharing procedure. Intuitively, as the local data set of a party *m* grows, it will reach a point were additional (synthetic) data from other parties will not have a strong effect on the analysis party *m* performs. Why then should that party participate and share its own data?

**Fig. 4:**
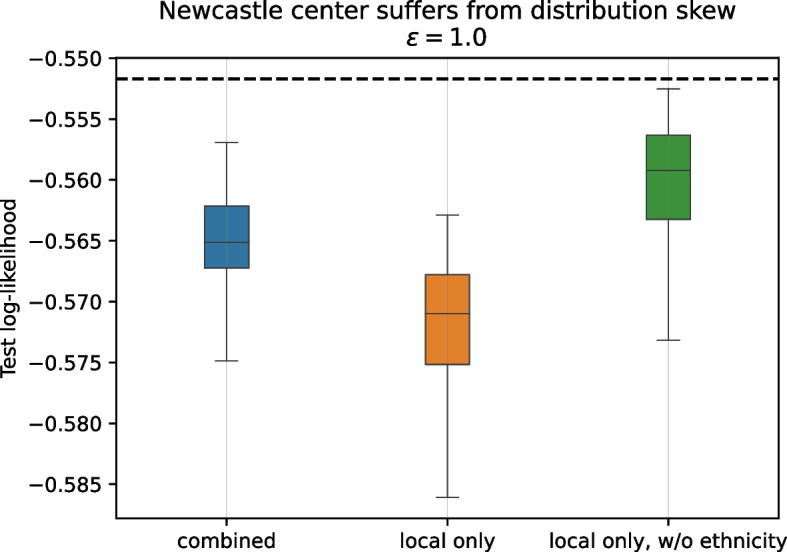
Training the analysis model on only *Newcastle*’s local data (full data without subsampling) results in poor predictive performance on global data due to skew in the local distribution (orange). Not considering the *ethnicity* feature when training on local data improves predictive performance (green). Combining local data with synthetic data also improves model performance while still considering all features, i.e., without need to change the model (blue). The dashed line indicates the log-likelihood of a model trained on the full population. Ten independent repeats. Observed pairwise differences between the means of the distributions are statistically highly significant ($p < 0.001, n_{\text {local only}}={1\,000}, n_{\text {combined}} = {100\,000}$)

To investigate, we isolate the largest assessment center of the UK Biobank data set, *Newcastle*, with ${4\,737}$ records in the full training set. Figure 4 shows that contrary to the argument made above, the log-likelihood of the analysis model on the global test set (blue box-plot) is higher than that for the model trained only using Newcastle’s local training data (orange).

#### *Newcastle* center is negatively affected by data skew

A plausible explanation for this is that the local data is not representative of the global population. It turns out that this particular center is much more ethnically homogeneous, consisting of 96.54 % records labelled *White British* compared to 88.28 % in the full UK Biobank cohort. This also manifests in a deviation of the local two-way marginal for the (*ethnicity*, *SARS-CoV-2 test result*) features from the population distribution (cf. Table S3).

To determine whether it is this skew that results in the comparatively poor performance of *Newcastle*, we train the analysis model on the center’s local data without taking ethnicity into consideration as a predictor. This model achieves better predictive accuracy (higher log-likelihood) on the global population (green box-plot of Fig. 4), indicating that this skew in the ethnic composition of the local data does result in an observable effect on global predictions. To learn a good analysis model, the center should therefore not adjust its model for ethnicity when training only on local data. However, this means that the model can no longer capture the statistical effect of ethnicity on the outcome. Incorporating shared data from other parties may be able to alleviate the skew in ethnicity, meaning that a model trained on that data would be able to capture its statistical effect. Figure 4 shows that the predictive accuracy of the analysis model trained using shared synthetic data also improves over just using the local data. In this case using combined data achieves a mean log-likelihood slightly worse than the model trained on local data only without considering ethnicity, but has the additional advantage that the model does not have to be modified.

#### Data sharing mitigates local distribution skew

To further assess the effect of different magnitudes of local skew, we introduce an artificial new party using half the data from the previously held-out test set. This party’s data therefore exactly matches the population distribution and consists of more data points (${5\,828}$) than any of the original parties. We then fix a category of the *ethnicity* feature, *South Asian*, and discard data points corresponding to the (*South Asian*, *positive SARS-CoV-2 test result*) marginal until only a fraction of $10\%, 25\%, 50\%, 75\%$ remains, respectively, to introduce different amounts of skew.^6^ This experimental set-up corresponds to the effect of minority groups being potentially less likely to report infections, e.g., due to fear of disadvantageous treatment or cases where systematic biases affect the collected data. We evaluate the predictive log-likelihoods on the *South Asian* subgroup of the remaining half of the test set to assess the resulting performance of the analysis model for that particular subgroup.

**Fig. 5:**
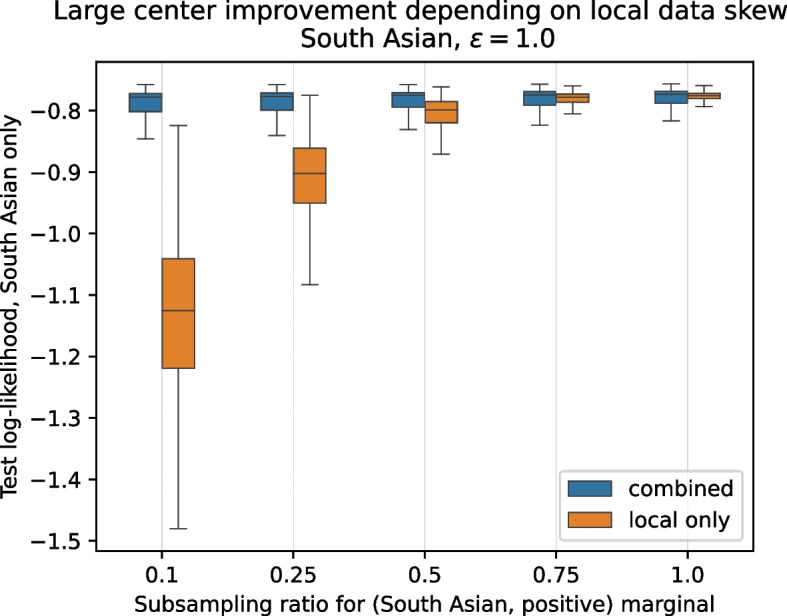
By participating in the data sharing, parties with large amounts of local data can correct for local skews in data distribution, with stronger corrective effects for stronger skews (corresponding to a smaller value on the x-axis). Observed differences in means are statistically highly significant ($p < 0.001, n_{\text {local only}} = {1\,000}, n_{\text {combined}} = {100\,000}$) for all skews

Figure 5 shows the predictive log-likelihood distribution for the model using only the (skewed) local data (orange) compared to incorporating shared synthetic data (blue) for the different levels of local skew. We observe that the stronger the skew (i.e., the smaller the subsampling ratio of the two-way marginal), the larger the corrective effect from incorporating synthetic data. On the other hand, if only small or no skew is present, the party may not benefit from participating in the sharing. Both of these observations are in line with our expectations.

## Discussion

In the above section we have empirically demonstrated on a real-world data set that data sharing using differentially private synthetic data from multiple sources improves the performance of the analysis task in many cases. We have particularly shown that the benefits from data sharing diminish more slowly than the deterioration of learning from only local data as the size of the local data set decreases. This is in contrast to the tentative argument made in the beginning that if the local data set is too small to solve the analysis task itself, it will also be too small to learn a good generative model, which would be required for synthetic data sharing to be useful. We believe the reasons for this apparent conflict are twofold: 1) The task faced by the generative model is to learn the *local distribution* of the data, which can be considerably easier, and 2) any remaining errors are sufficiently mitigated by combining synthetic data from multiple sources. This is good news, as it allows data sharing to be adopted in the low data regime, for example, to perform analyses early in an initial data collection phase when only few data points have yet been accumulated at any particular site. The onset period of the SARS-CoV-2 pandemic is an example of exactly such a situation.

We have further seen that the most drastic increase in performance is obtained already with only a small number of parties (5) participating. Additional data from more sources will likely not improve the results much further. This is encouraging as it suggests that no large consortium of parties is needed to benefit from the data sharing approach, and any party starting to share their data can do so with the knowledge that only a few like-minded parties are required to reap benefits for all. However, if less than three synthetic data sets are made available or if the local data is already a good representation of the global population statistics, there is a risk of low quality synthetic data having a negative impact on the analysis. We discuss this point further in the section entitled Synthetic data does not unconditionally improve analysis.

Finally, we have experimentally confirmed that parties can successfully correct biases in their data that arise from a local skew of the data distribution, such as the misrepresentation of a minority group. This holds even for the case of parties that already have a large data set, incentivising them to participate and share their data as well.

In the following sections we discuss variations to the assumptions we made for our experiments (Section Validity of assumptions), previous results in the literature about potential harmful effects of using synthetic data (Section Synthetic data does not unconditionally improve analysis), and whether parties could assess whether their analysis improves (Section Can parties evaluate whether shared data improves their analysis model?). We conclude with a brief discussion of the effect of the privacy parameters (Section Effect of the privacy parameter) and the Federated Learning methodology as a related popular approach for learning from distributed data (Section Federated learning).

### Validity of assumptions

In this work we have made several assumptions that we stated in the introduction. We now briefly discuss some possible relaxations and how they would likely affect the results presented above.

We make the simplifying assumption that all parties aim to optimise their analysis on the population level rather than for their local data distribution. If a party instead prefers to optimise for the local distribution there is a high chance that incorporating shared data from other parties decreases this performance (by shifting the training data more towards the overall population distribution) - provided that the party’s local distribution deviates from the overall population. On the other hand, for very small local data, the overall population might still add valuable information to aid the learning of the local analysis model. The strength of either effect would likely depend on the amount of deviation and the relative amount of data obtained from other parties. However, the party could easily check whether shared data would improve their analysis by participating in the sharing and simply testing the result on a held-out portion of their data.

We have also assumed that the results of a party’s analysis are kept private, i.e., no potential privacy leakage can occur from performing the analysis. If the analysis result is intended to be published, additional measures have to be taken to ensure that the usage of the party’s local data does not leak additional data. This could be, e.g., achieved by employing differential privacy in the analysis task or substituting the party’s own synthetic data in place of its actual data. However, we consider this as an orthogonal problem.

Another assumption we made is that all parties reciprocate in sharing, i.e., if they want to use shared data from other parties, they will also share synthetic data derived from their own data. This seems essential for a fair distribution of the burden of making data available and could be enforced by each party licensing their shared data under conditions that require other parties using it to share alike. However, it is not a strict requirement as long as there is a sufficient number of parties willing to share their data for any reason. Fortunately, as we have seen in the experiments, that number can be quite small and still allow for everyone to see drastic benefits.

We also assumed parties are non-malicious and will not publish data engineered to negatively affect the performance of other parties. Following our procedure a malicious party could sample a large set of arbitrarily bad artificial data to poison the well for all other parties. However, given a sufficient number of non-malicious parties sharing, it is likely that the other parties could filter out such bad shared data sets by comparison with other shared data sets, resulting in some robustness of the overall approach. We consider this to be an interesting future direction.

Finally, we made the implicit assumption that the parties are in agreement over the data features to share and how they are to be represented in digital tabular form. We consider this a minor obstacle: Since the approach we consider results in each party making a synthetic data set available, any other party can simply adopt the format of data sets already published, or convert those to its own representation in a preprocessing step, without the need for explicit coordination between parties. Furthermore, parties are not bound to all include the same exact set of features in the synthetic data they publish; but a downstream analysis that requires a certain set of predictors would then have to exclude data from parties that are missing relevant features. For our case study, we consider this problem as orthogonal, but recognise it as an interesting avenue for future work with potential links to existing literature on data integration and learning from incomplete data.

### Synthetic data does not unconditionally improve analysis

Strong privacy protection comes with a trade-off in the usability of the synthetic data. The more privacy we require, the less details we can learn from the data. This trade-off is more severe when data sets are small, because then each individual record has larger effects on the statistics learned from the data, forcing privacy-preserving methods to put stronger restrictions on the learned signals. Hence, statistical signals of the original data tend to become weaker in the synthetic data. Additionally, learning a generative model often involves approximations, which can also limit the statistical signal captured in the synthetic data. Sampling a finite synthetic data set further introduces uncertainty about the learned parameters of the generative model.

As a final further complication particular to our setting, the local data contributed by another party could follow a distribution skewed away from that of the overall population - this skew then transfers to the synthetic data. Summarising, there are three main factors that can cause synthetic data to be of low quality: 1) the underlying data set was too small to learn a good generative model under differential privacy, 2) the data was skewed away from the population distribution, and, 3) the chosen generative model results in loss of information or skew. Hence, parties interested in the underlying sensitive data might be reluctant to use synthetic data for fear of obtaining bad data.

Wilde et al. [13] showed that combining data with a single synthetic data set may lead to worse utility in statistical analysis. Our experiments corroborate their findings and show a large spread in log-likelihood distribution for the analysis task when using data shared by only one or two sources (cf. Section Gains increase quickly with number of shared data sets): In this case there is a relatively high chance of falling victim to a skewed synthetic data set which harms performance of the analysis task compared to only using local data. However, as synthetic data from more and more other centers is combined, the impact of any single bad data set is reduced and we see consistent improvements up to a certain limit prescribed by the privacy constraints. This is likely because the data underlying the synthetic data becomes a more accurate representation of the population, which eliminates error caused by shift of the local distribution at different centers (Error Source 2). Additional reduction of error may result from averaging out the (largely) independent errors from sources 1) and 3) across multiple synthetic data sets and a general increase in size of available data, although further work is required to confirm this.

As detailed in Section Sharing multiple synthetic data sets we additionally rely on repeated sampling of synthetic data and application of Rubin’s rules [32, 33] to quantify the additional uncertainty introduced by sampling finite (small) data sets from the learned generative models at no additional privacy cost.

However, as pointed out by Wilde et al. [13], for strict levels of privacy, relying on large amounts of synthetic data may still lead to worse results if the local data was already a representative sample of the overall population. However, for practical levels of privacy parameter $\varepsilon$, this error appears to be small in our experiments.

### Can parties evaluate whether shared data improves their analysis model?

One of our assumptions in this paper is that all parties aim to optimise the performance of their analysis on the global population. To evaluate this we have used a test set corresponding to the population distribution that we separated from the data prior to running our experiments. However, in practice, parties generally do not have access to an unbiased sample from the population but only their local data. This means it is not trivial for a party to test whether using shared data actually improves their analysis. Testing with a held-out portion of the local data can mislead the party if the local data is skewed away from the population, which the party cannot know a-priori.

As a potential solution to this, parties could establish a joint testing protocol which informs a party of the test performance of their analysis model on population data. This may be based on techniques from the literature on secure multi-party computation [37, 38] and differential privacy to safeguard learned models and data in the process. Alternatively, an approach using parts of the shared data as a proxy for the population data in testing could be feasible as well. We consider solutions for this an important aspect for future work; here we did not take a stance on what of the alternative solutions is used, and report results assuming it had been solved.

### Effect of the privacy parameter

The amount of privacy protection afforded by differential privacy is controlled by a privacy parameter $\varepsilon$ (cf. Section Differentially private data sharing). All the results shown above were achieved for a fixed amount of $\varepsilon =1$ to achieve comparable results across the different experiments. A stronger level of privacy ($\varepsilon < 1$) results in a reduction of the information captured in the synthetic data and vice-versa. While this decreases analysis performance achieved by the data sharing approach compared to the impractical full pooling of data, a gradual change in $\varepsilon$ does not fundamentally change the overall outcomes and trends we have observed in this paper.

**Fig. 6:**
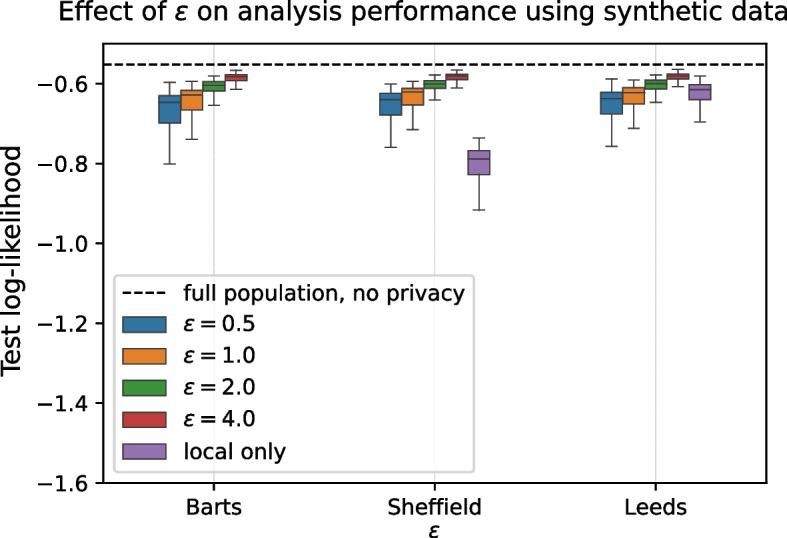
Tighter privacy parameter $\varepsilon$ gradually decreases the information captured in the shared synthetic data. Shown is the log-likelihood of the analysis model when combining local data with shared synthetic data from all other centers for different values of privacy parameter $\varepsilon$. For $\varepsilon =1$, the results are the same as in as in Fig. 1. For comparison results for performing the analysis exclusively on local data (“local only”) are also included (in that case no data is shared). Local only result for *Barts* cut off for readability

Figure 6 demonstrates this for four different values of $\varepsilon$ (0.5, 1, 2, 4) for the same three representative centers as in Section Gains increase quickly with number of shared data sets. It shows the predictive log-likelihood after combining local data with shared synthetic data from all other centers, as in Fig. 1. Results for performing the analysis on only the locally available data are also included for comparison, but we stress that in this case no data is being shared amongst the parties (giving an effective $\varepsilon$ of 0). Notably, we see that a modest increase in $\varepsilon$ to 2 improves the result even for *Leeds* over using local data. We include variants of Figs. 1 and 2 for this privacy level in the supplement as Figs. S1 and S2, which demonstrate that in this case all parties consistently benefit from participating in the data sharing approach.

### Federated learning

Federated learning (FL) [14] is another collaborative learning framework where the aim is to learn from distributed data without explicitly combining the data of the parties (typically named *clients* in FL). The clients collaboratively learn a model by locally computing model updates using their own data and sending the updates to an aggregation server which then updates the model’s parameters and shares the updated model with the clients. While standard FL does not provide any formal privacy guarantees, there are works merging FL with DP [39–41]. Although FL (or its DP variants) can provide good performance for specific, predetermined tasks, it still lacks the generality that synthetic data sharing provides: The data can be used in arbitrary future tasks without any further computational effort or additional expenditure of privacy budget by the parties. FL also requires non-trivial infrastructure: It typically needs a central coordinating party as well as secure real-time two-way communication between that coordinator and all other parties for iterative updates. Our data sharing procedure requires no explicit coordination with other parties, i.e., each party can prepare their synthetic data in a completely *offline* fashion. We only assume that each party has a way of making their synthetic data available to other parties and obtaining synthetic data previously published by the other parties, asynchronously. An important benefit of this is that a party joining late does not necessitate actions from the other parties: These may retrain their local analysis model based on the added synthetic data if they wish to, but they do not need to retrain their generative model or publish new data. In contrast, FL approaches would require to either restart the whole procedure or employ some federated model updating procedure.

However, in cases where combined data is only ever used in a single, well-defined task, an FL approach will likely result in a better privacy–utility trade-off as it can expend all of the available privacy budget on learning that task well. In contrast, our data-sharing-based approach requires each party to learn a generative model that captures the complete (local) data distribution. FL is also likely preferable for very small local data sets, for which learning a generative model is infeasible.

There have also been proposals for federated learning that generate data points akin to coresets [42] as part of the iterative updating process, notably [43]. While [43] refer to these data points as synthetic data, they typically do not resemble the original data and have no meaningful semantic interpretation. This is because they are only intended for internal use in their FL algorithm, which outputs parameters for a particular model and not a synthetic data set. These methods also have the same overall structure as prior FL algorithms and hence the same coordination requirements.

## Conclusions

In this work we have empirically investigated the practical feasibility of collaborative learning under privacy constraints from shared synthetic data prepared by non-coordinated parties. We observe that given a sufficient amount of available synthetic data sets, almost all involved parties in general obtain better results in their analysis task compared to using only their locally available data. This is true even if individual synthetic data sets are based on small amounts of local data and may be of poor quality. While our experiments also corroborate earlier findings that relying too much on a single synthetic data set can negatively impact the analysis, we find our overall results encouraging: They suggest that making synthetic data widely available can help overcoming data scarcity issues for privacy sensitive domains as well as the aforementioned quality impacts of relying on single synthetic data sets, while posing only weak assumptions on an individuals party’s ability or willingness to coordinate with other parties. This is in contrast to federated learning approaches, which may be able to achieve better utility on case-by-case basis but require a great deal of coordination between parties.

While in our study we considered only a single data set as well as method for DP synthetic data generation, we stress that the overall setting of collaboration by means of publishing synthetic data sets is not restricted to this particular context. We expect our results to generalise for other combinations of data sets and synthetic data generation approaches. An interesting case that we did not explore in this paper is the setting when the parties are interested in applying different analysis tasks and each might tailor their synthetic data to their particular task. We consider this for future work as a natural extension of our present study, in addition to those avenues already pointed in the Discussion.

### Supplementary Information

Supplementary Material 1.

## Data Availability

The code to run the experiments can be found on https://github.com/DPBayes/Collaborative-Learning-with-DP-Synthetic-Twin-Data. The data used is available from the UK Biobank upon request but subject to a screening process of the intended study as well as an access fee.

## Abbreviations

DP: Differential Privacy
FL: Federated Learning

## Footnotes

1 The exact data fields we used from the UK Biobank repository are listed in Table S1.

2 A more detailed listing of center sizes can be found in Table S2.

3 The mean is taken element-wise.

4 Test data is held-out, i.e., the local data $D_m$ used by party *m* for training generative and analysis models (*G*, *f*) excludes $D_{m,\text {test}}$ in the experiments.

5 100 - 500 data points per center after sampling.

6 In mathematical terms, let $D_{\text {South Asian, positive}} \subset D_m$ denote the subset of data points with *ethnicity* as *South Asian* and a *positive* SARS-CoV-2 test result. $D_{\text {South Asian, negative}} \subset D_m$ is correspondingly the subset of those with *negative* test result. We introduce skew by subsampling $D_{\text {South Asian, positive}}$ with probability $p \in \{0.1, 0.25, 0.5, 0.75, 1\}$ while leaving $D_{\text {South Asian, negative}}$ unchanged.
