# Supplementary material for: Collaborative learning from distributed data with differentially private synthetic data
Source: BMC Med Inform Decis Mak. 2024 Jun 14;24:167. doi: 10.1186/s12911-024-02563-7 (PMC11179391; doi:10.1186/s12911-024-02563-7)
Supplement: Supplementary file 1 — Supplementary Material 1. [file 12911_2024_2563_MOESM1_ESM.pdf]

Table S1: Data fields of the UK Biobank used for this work.

Field ID	Field title	Purpose
31	Sex	analysis predictor
54	UK Biobank assessment centre	analysis parties
189	Townsend deprivation index	analysis predictor
6138	Qualifications	analysis predictor
21000	Ethnic background	analysis predictor
21022	Age at recruitment	analysis predictor
40000[1]	Date of death	pre-processing (filtering)
40100[2]	Records of SARS-CoV-2 test results	analysis target

[1]Used to filter participants. Filtering and binning of data prior to running the experiments was following the preprocessing steps of Niedzwiedz et al. [21].

[2] When data were obtained, SARS-CoV-2 test results were instead made available as a data table alongside but separate of the main UK Biobank repository, cf. https://biobank.ctsu.ox.ac.uk/crystal/exinfo.cgi?src=COVID19.

Table S2: Assessment center data set sizes.

Assessment center	Total size	Training size
Newcastle	5 922	4 737
Bristol	5 860	4 688
Reading	4 479	3 583
Leeds	4 424	3 539
Bury	4 345	3 476
Nottingham	4 236	3 388
Hounslow	3 984	3 187
Liverpool	3 946	3 156
Croydon	3 513	2 810
Birmingham	3 271	2 616
Sheffield	3 042	2 433
Middlesborough	2 857	2 285
Stoke	2 715	2 172
Barts	1 918	1 534
Manchester	1 874	1 499
Oxford	1 867	1 493

Table S3: Two-way marginals for ethnicity and SARS-CoV-2 test result, full cohort and *Newcastle* center.

Ethnicity	Covid Test	Full Cohort	Newcastle
White British	neg.	19.94	18.89
	pos.	68.33	77.64
White Other	neg.	2.38	0.92
	pos.	0.73	0.14
White Irish	neg.	2.04	0.88
	pos.	0.61	0.19
South Asian	neg.	1.33	0.36
	pos.	0.94	0.17
Black	neg.	1.20	0.08
	pos.	0.94	0.06
Other	neg.	0.62	0.19
	pos.	0.34	0.08
Mixed	neg.	0.41	0.27
	pos.	0.19	0.04
Chinese	neg.	0.14	0.04
	pos.	0.05	0.0

All values given in per cent. *Newcastle*'s two-way marginals are significantly different to those of the full cohort.

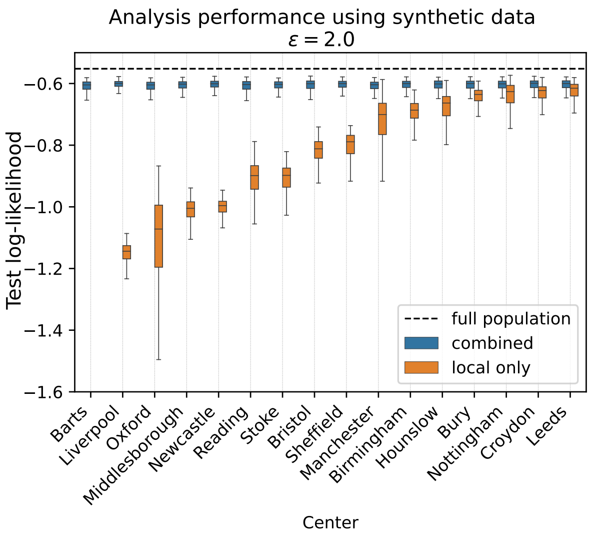

Fig. S1: Right side of Fig. 1 repeated with privacy parameter $\varepsilon = 2$: Higher ε, i.e., less strict privacy guarantees, allows synthetic twin data to carry more information from underlying sensitive data. This reflects in better performance of the model trained with combined data, both in terms of location and spread of the predictive log-likelihood on the test set. All centers now benefit from combining their local data with shared synthetic data.

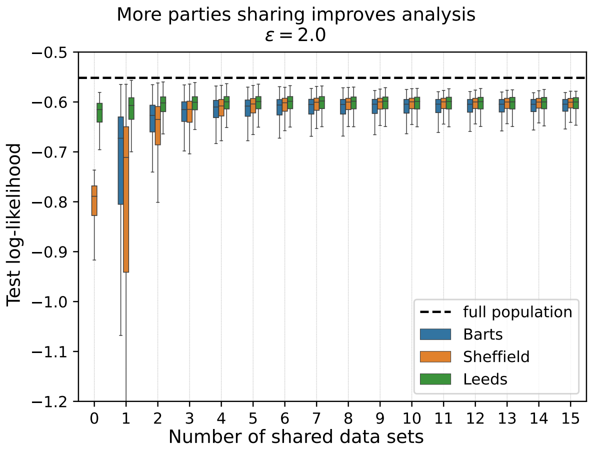

Fig. S2: Fig. 2 repeated with privacy parameter $\varepsilon = 2$: Higher ε, i.e., less strict privacy guarantees, allows synthetic data to carry more information from underlying sensitive data. Improvements from using more data from additional centers are more pronounced and continue throughout.

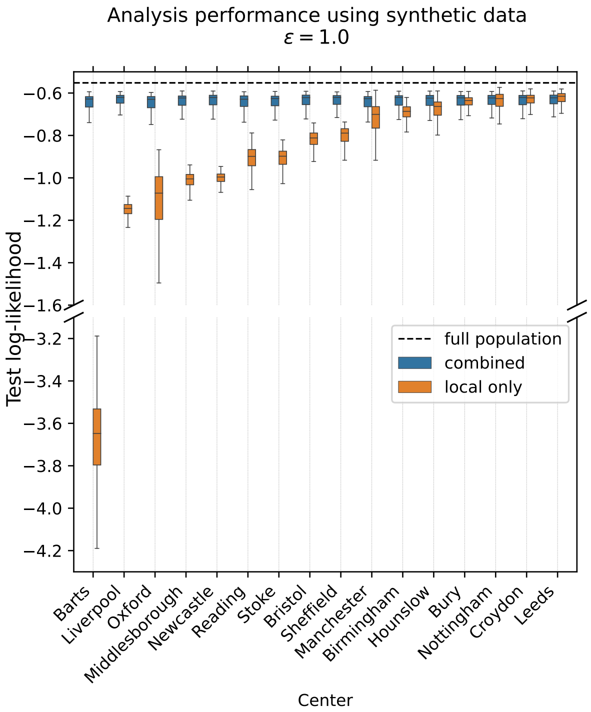

Fig. S3: Full version of right side of Fig. 1.

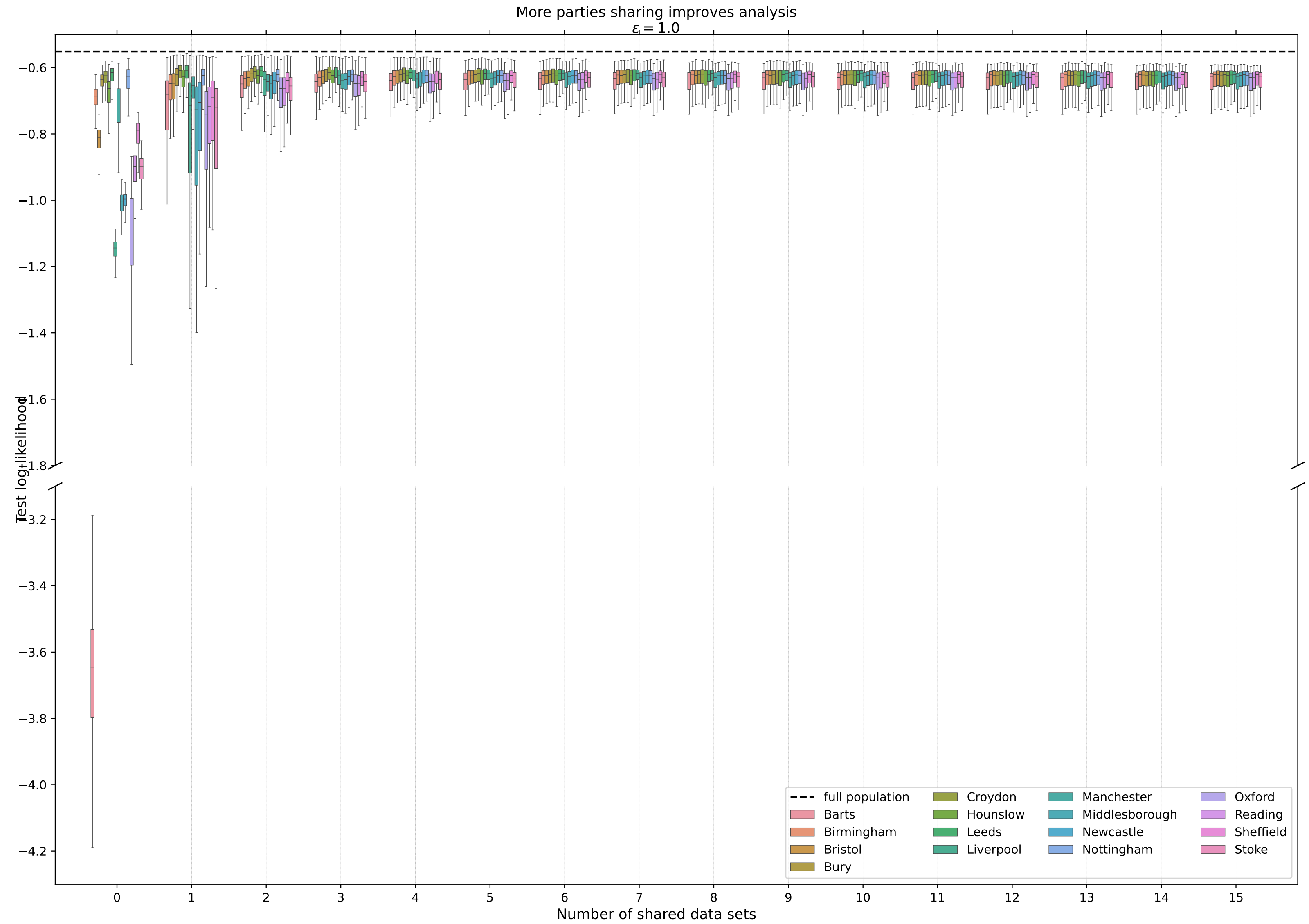

Fig. S4: Full version of Fig. 2: The log-likelihood of the learned model increases rapidly as synthetic data from other centers becomes available. Spread in log-likelihoods may initially increase when only a few synthetic data sets are incorporated, but then diminishes rapidly with the number of additional data sets. The dashed black line shows the log-likelihood for an ideal setting where the analysis could be performed over the combined data of all parties. 100 repeats with different orders in which synthetic data is added and 10 repeats of releasing synthetic data.

Table S4: p-values for improvements in log-likelihood mean score when adding synthetic data from an additional center.

local center	$0 \to 1$	$\to 2$	$\to 3$	$\to 4$	$\to 5$	$\to 6$	$\to 7$	$\to 8$	$\to 9$	$\to 10$	$\to 11$
Barts	***	***	***	***	***	***	***	0.752	0.39	0.962	0.75
Birmingham	***	***	***	***	***	***	0.007**	1.0	1.0	1.0	1.0
Bristol	***	***	***	***	0.931	0.002**	0.014*	1.0	0.999	1.0	1.0
Bury	***	***	1.0	1.0	1.0	1.0	1.0	1.0	1.0	1.0	1.0
Croydon	***	1.0	1.0	1.0	1.0	1.0	1.0	1.0	1.0	1.0	1.0
Hounslow	***	***	0.539	1.0	1.0	0.999	0.981	1.0	0.987	1.0	1.0
Leeds	***	1.0	1.0	1.0	1.0	1.0	1.0	1.0	1.0	1.0	1.0
Liverpool	***	***	***	***	***	0.009**	0.952	1.0	1.0	1.0	1.0
Manchester	***	***	***	***	***	0.001**	0.111	0.986	***	0.83	0.967
Middlesborough	***	***	***	***	***	***	***	0.998	0.568	0.999	1.0
Newcastle	***	***	***	***	***	0.905	1.0	1.0	0.985	1.0	1.0
Nottingham	0.003**	***	1.0	1.0	1.0	1.0	0.999	1.0	0.891	1.0	1.0
Oxford	***	***	***	***	***	***	***	0.781	***	0.066	0.274
Reading	***	***	***	***	***	***	***	0.451	***	0.091	0.281
Sheffield	***	***	***	***	***	***	0.98	1.0	0.995	1.0	1.0
Stoke	***	***	***	***	***	***	***	0.542	0.012*	0.67	0.887

Each row shows the perspective of a local center. Each column reports p-values for the one-sided test that adding one more center's data improves the previous mean log-likelihood. Significance levels are $p \leq 0.001$ (***), $p \leq 0.01$ (**) and $p \leq 0.05$ (*). Columns $11 \to 12$ to $\to 15$ with all values > 0.5 omitted due to space constraints. $n = 100\,000$.
